# Serum resistin levels in critically ill patients are associated with inflammation, organ dysfunction and metabolism and may predict survival of non-septic patients

**DOI:** 10.1186/cc7925

**Published:** 2009-06-19

**Authors:** Alexander Koch, Olav A Gressner, Edouard Sanson, Frank Tacke, Christian Trautwein

**Affiliations:** 1 Department of Medicine III, RWTH-University Hospital Aachen, Pauwelsstrasse 30, 52074 Aachen, Germany; 2 Institute of Clinical Chemistry and Pathobiochemistry, RWTH-University Hospital Aachen, Pauwelsstrasse 30, 52074 Aachen, Germany

## Abstract

**Introduction:**

Blood glucose levels and insulin resistance in critically ill patients on admission to intensive care units (ICUs) have been identified as factors influencing mortality. The pathogenesis of insulin resistance (IR) in critically ill patients is complex and not fully understood. Resistin is a hormone mainly derived from macrophages in humans and from adipose tissue in rodents, which regulates glucose metabolism and insulin sensitivity. In non-critically ill patients, resistin was found to be related to impaired glucose tolerance, insulin resistance, metabolic syndrome, obesity and type 2 diabetes. Therefore, resistin might represent a link between inflammation, acute phase response and insulin resistance in critically ill patients. We aimed to examine the correlation of serum resistin concentrations to parameters of inflammation, organ function, metabolism, disease severity and survival in critically ill patients.

**Methods:**

On admission to the Medical ICU, 170 patients (122 with sepsis, 48 without sepsis) were studied prospectively and compared with 60 healthy non-diabetic controls. Clinical data, various laboratory parameters, metabolic and endocrine functions as well as investigational inflammatory cytokine profiles were assessed. Patients were followed for approximately three years.

**Results:**

Resistin serum concentrations were significantly elevated in all critical care patients compared with healthy controls, and significantly higher in sepsis than in non-sepsis patients. Serum resistin concentrations were not associated with pre-existing type 2 diabetes or obesity. For all critically ill patients, a correlation to the homeostasis model assessment index of insulin resistance (HOMA-IR) was shown. Serum resistin concentrations were closely correlated to inflammatory parameters such as C-reactive protein, leukocytes, procalcitonin, and cytokines such as IL6 and TNF-α, as well as associated with renal failure and liver synthesis capacity. High resistin levels (> 10 ng/ml) were associated with an unfavourable outcome in non-sepsis patients on ICU and the overall survival.

**Conclusions:**

Serum resistin concentrations are elevated in acute inflammation due to sepsis or systemic inflammatory response syndrome (SIRS). The close correlation with other acute phase proteins suggests a predominant, clinically relevant resistin release from macrophages in ICU patients. Moreover, resistin could potentially serve as a prognostic biomarker in non-sepsis critically ill patients.

## Introduction

Hyperglycemia, impaired glucose tolerance and insulin resistance are common findings in critically ill patients with sepsis or septic shock [1,2]. Maintenance of normoglycemia (blood glucose levels ≤ 110 mg/dL) by intensive insulin therapy improves survival and reduces morbidity in critically ill patients after cardiac surgery [3]; nevertheless its impact on the outcome of patients in medical intensive care units (ICU) is an ongoing matter of debate, especially with regard to the safety of tight blood glucose control and the effectiveness in this cohort [4,5]. In patients with obesity, metabolic syndrome and type 2 diabetes, characterized by target-tissue resistance to insulin, adipocyte-derived factors (adipokines) have been identified which signal to the brain, adipose tissue, liver, muscle, and the immune system, and thus influence insulin resistance [6]. Obesity itself is regarded as a proinflammatory state with oxidative stress showing increased levels of TNF-α, IL-6, and C-reactive protein (CRP) [7]. The mechanisms of insulin resistance in the clinical setting of severe sepsis are numerous and not exactly understood [8].

Identifying novel biomarkers for linking these states of acute and subacute inflammation with metabolism is crucial for further risk stratification of critically ill and septic patients in the ICU and developing new therapeutic strategies. Resistin (named for resistance to insulin) has been proposed as a novel marker of inflammatory response in sepsis. This is because elevated resistin plasma levels were found in patients with severe sepsis and septic shock and were associated with severity of disease as measured by Acute Physiology and Chronic Health Evaluation II (APACHE II) score; however, a correlation to patient outcome and survival could not be demonstrated [9].

In 2001, resistin was originally reported as an adipose tissue-specific hormone. In animal models resistin is clearly linked to obesity, metabolic syndrome and type 2 diabetes, in which hyperglycemia and hyperinsulinemia increase resistin expression [10]. Murine resistin is primarily produced in adipocytes, whereas resistin in humans is mainly derived from macrophages rather than adipocytes [11,12]. Furthermore, the protein sequences of murine and human resistin are only approximately 60% identical. This was thought to contribute to the fact that data from animal models could be only partially translated to humans [13-15], leaving the role of resistin in humans less certain and suggesting varying physiologic relevances in both human and rodent systems.

A recent study, using a novel 'humanized resistin mouse' model that lacks adipocyte-produced mouse resistin but expresses human resistin derived from macrophages, could show that macrophage-derived human resistin contributes to insulin resistance by means of its inflammatory properties [16].

In the present study, we investigated serum resistin concentrations in a large cohort of critically ill patients in a medical ICU to understand the regulation of resistin with respect to inflammation, infection, hyperglycemia, and insulin resistance in critically ill patients and its potential use as a biomarker in ICU patients.

## Materials and methods

### Study design and patient characteristics

We studied 170 patients (111 male, 59 female with a median age of 63 years; range 18 to 86 years) who were admitted to the General Internal Medicine ICU at the RWTH-University Hospital Aachen, Germany (Table 1). The study protocol was approved by the local ethics committee and written informed consent was obtained from the patient, his or her spouse, or the appointed legal guardian. Patients that were expected to have a short-term (< 72 hours) intensive care treatment due to post-interventional observation or acute intoxication were not included in this study. Medium length of stay at the ICU was 8.5 days (range 1 to 137 days) and medium length of stay in hospital was 27 days (range 2 to 151 days).

**Table 1:** Characteristics of the study population

**Parameter**	**All ICU patients**	**Sepsis**	**Non-sepsis**
Number	170	122	48
Sex (number male/number female)	111/59	81/41	30/18
Sex (% male/female)	65/35	66/34	62/38
Age median (years; range)	63 (18 to 86)	64 (20 to 86)	59.9 (18 to 79)
BMI median (range)	25.8 (14 to 59.5)	25.99 (14 to 59.5)	25.1 (17.5 to 53.3)
Median days ICU (range)	8.5 (1 to 137)	10 (1 to 137)	6 (1 to 45)
Median days hospital (range)	27 (2 to 151)	30 (2 to 151)	14 (2 to 85)
Death ICU n (%)	54 (31.8)	42 (34.4)	12 (25)
Survival ICU n (%)	116 (68.2)	80 (65.6)	36 (75)
Death follow-up n (%)	88 (51.8)	64 (52.5)	24 (50)
Survival follow-up n (%)	82 (48.2)	58 (47.5)	24 (50)
Ventilation, n (yes/no)	113/57	82/40	31/17
Median ventilation time hours (range)	66 (0 to 2966)	127.5 (0 to 2966)	31 (0 to 755)
Median CRP (mg/dl; range)	90.5 (5 to 230)	129.5 (5 to 230)	14.5 (5 to 164)
Median creatinine (mg/dl; range)	1.7 (0.1 to 14.1)	1.9 (0.1 to 14.1)	1.3 (0.3 to 13.1)
Median cystatin C (mg/l; range)	1.83 (0.41 to 7.30)	1.98 (0.41 to 6.33)	1.34 (0.41 to 7.30)
Median lactate (nmol/l; range)	1.7 (0.4 to 21.9)	1.7 (0.4 to 21.9)	2.1 (0.7 to 18.1)
Median APACHE II score (range)	14 (0 to 31)	14 (0 to 31)	15 (0 to 31)
Median SAPS-2 score (range)	44 (0 to 80)	45 (0 to 79)	41 (13 to 80)

APACHE = Acute Physiology and Chronic Health Evaluation; BMI = body mass index; CRP = C = reactive protein; ICU = intensive care unit; SAPS = simplified acute physiology score.

Patient data, clinical information and blood samples were collected prospectively. The clinical course of patients was observed in a follow-up period by directly contacting the patients, the patients' relatives or their primary care physician over a period of about 900 days. Critical care patients were divided into two categories: sepsis patients and non-sepsis patients. Patients in the sepsis group met the criteria proposed by the American College of Chest Physicians and the Society of Critical Care Medicine Consensus Conference Committee for severe sepsis and septic shock [17].

The control group consisted of 60 healthy non-diabetic blood donors (33 male, 27 female, with a median age of 51 years; range 31 to 69 years) with normal values for blood counts, CRP, and liver enzymes.

### Characteristics of sepsis and non-sepsis patients

Among the 170 critically ill patients enrolled in this study, 122 patients conformed to the criteria of bacterial sepsis (Table 1). In the majority of sepsis patients the identified origin of infection was pneumonia (Table 2). Non-sepsis patients did not differ in age or sex from sepsis patients and were admitted to the ICU due to cardiopulmonary disorders (myocardial infarction, pulmonary embolism, and cardiac pulmonary edema), decompensated liver cirrhosis, or other critical conditions. Sepsis patients more often required mechanical ventilation in the longer term compared with the non-sepsis patient group (Table 1). As expected significantly higher levels of laboratory indicators of inflammation (i.e. CRP, procalcitonin, white blood cell count) were found in sepsis patients (Table 1, and data not shown). Nevertheless, both groups did not differ in APACHE II score, vasopressor demand, or laboratory parameters indicating liver or renal dysfunction (data not shown). Among all critical care patients, 32% died in the ICU, and an additional 52% of the total initial cohort died during the overall follow-up of 900 days. In sepsis and non-sepsis patients no significant differences in rates of death and survival were observed.

**Table 2:** Disease etiology of the study population

	**Sepsis**	**Non-sepsis**
	n = 122	n = 48
**Etiology of sepsis critical illness** Site of infection n (%)		
Pulmonary	72 (59%)	
Abdominal	22 (18%)	
Other	28 (23%)	
**Etiology of non-sepsis critical illness** n (%)		
Decompensated liver cirrhosis		17 (35%)
Cardiopulmonary diseases		18 (38%)
Other		13 (27%)

### Comparative variables

The patients in the sepsis and non-sepsis groups were compared by age, sex, body mass index (BMI), pre-existing diabetes mellitus, and severity of disease using the APACHE II score [18] at admittance.

ICU treatment such as volume therapy, vasopressor infusions, demand of ventilation and ventilation hours, antibiotic and antimycotic therapy, renal replacement therapy, and nutrition were recorded, alongside a large number of laboratory parameters that were routinely assessed during ICU treatment. Resistin serum concentrations were analysed using a quantitative sandwich immunoassay (ELISA; BioVendor, LLC, Candler, NC, USA). IL-6, IL-10, TNF-alpha (all Siemens Healthcare, Erlangen, Germany), and procalcitonin (Kryptor, B.R.A.H.M.S. Diagnostica, Henningsdorf, Germany) were measured by commercial chemiluninescence assays, following manufacturers' instructions.

### Statistical analysis

Due to the skewed distribution of most of the parameters, data are given as median, minimum, maximum, and 95% confidence interval. Differences between two groups are assessed by Mann-Whitney U test and multiple comparisons between more than two groups have been conducted by Kruskal-Wallis analysis of variance and Mann-Whitney U test for *post hoc *analysis. Box plot graphics illustrate comparisons between subgroups. They display a statistical summary of the median, quartiles, range, and extreme values. The whiskers extend from the minimum to the maximum value excluding outside and far-out values, which are displayed as separate points. An outside value (indicated by an open circle) is defined as a value that is smaller than the lower quartile minus 1.5-times interquartile range, or larger than the upper quartile plus 1.5-times the interquartile range. A far-out value is defined as a value that is smaller than the lower quartile minus three times interquartile range, or larger than the upper quartile plus three times the interquartile range [19]. All values, including outliers, have been included for statistical analyses. Correlations between variables have been analyzed using the Spearman correlation tests, where values of *P *< 0.05 were considered statistically significant. The prognostic value of the variables was tested by univariate and multivariate analysis in the Cox regression model. Kaplan-Meier curves were plotted to display the impact on survival. All statistical analyses were performed with SPSS version 12.0 (SPSS, Chicago, IL, USA).

## Results

### Resistin serum concentrations are elevated in all critical care patients and significantly higher in sepsis than in non-sepsis patients

**Figure 1:**
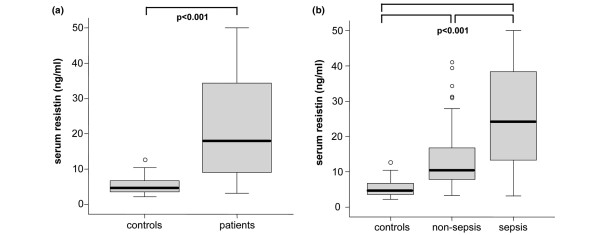
Serum resistin concentrations in critically ill patients. **(a) **Serum resistin levels are significantly (*P *< 0.001, U-test) elevated in all patients in the intensive care unit (n = 170) as compared with healthy controls (n = 60). **(b) **Serum resistin levels are significantly (*P *< 0.001, U-test) higher in sepsis patients (n = 122) as compared with non-sepsis (n = 48) patients. Box plots are displayed, where the bold black line indicates the median per group, the box represents 50% of the values, and horizontal lines show minimum and maximum values of the calculated non-outlier values; open circles indicate outlier values.

As demonstrated in Table 3 and Figure 1a critical care patients had significantly higher resistin serum levels than healthy volunteers in the control group (median 18 ng/ml in patients vs. 4.7 ng/ml in controls; *P *< 0.001). Resistin did not correlate with age or sex in either controls or patients (data not shown).

**Table 3:** Comparison between healthy volunteers and patients from the intensive care unit

	**Controls**	**All ICU patients**	**Sepsis**	**Non-sepsis**
	n = 60	n = 170	n = 122	n = 48
Resistin (ng/ml) median (range)	4.7 (2.2 to 12.7)	18 (3.22 to 50)	24.2 (3.22 to 50)	10.5 (3.33 to 41.1)
Resistin (ng/ml) 90%-interval	2.6 to 10.2	4.8 to 46.4	4.8 to 49.9	3.6 to 39.0

ICU = intensive care unit.

The subgroup analysis of septic and non-septic patients showed significantly higher resistin serum levels in the group of septic patients (median 24.2 ng/ml in patients with sepsis vs. 10.5 ng/ml in ICU patients without sepsis, *P *< 0.001; Figure 1b).

### Resistin serum concentrations are not correlated with pre-existing diabetes mellitus or BMI

Resistin has been initially identified as an adipocytokine related to insulin resistance, diabetes, and obesity [20]. To evaluate the effect of pre-existing diabetes mellitus and BMI we examined subgroups of diabetes patients and patients with BMI greater than 30 in the sepsis and non-sepsis cohorts.

**Figure 2:**
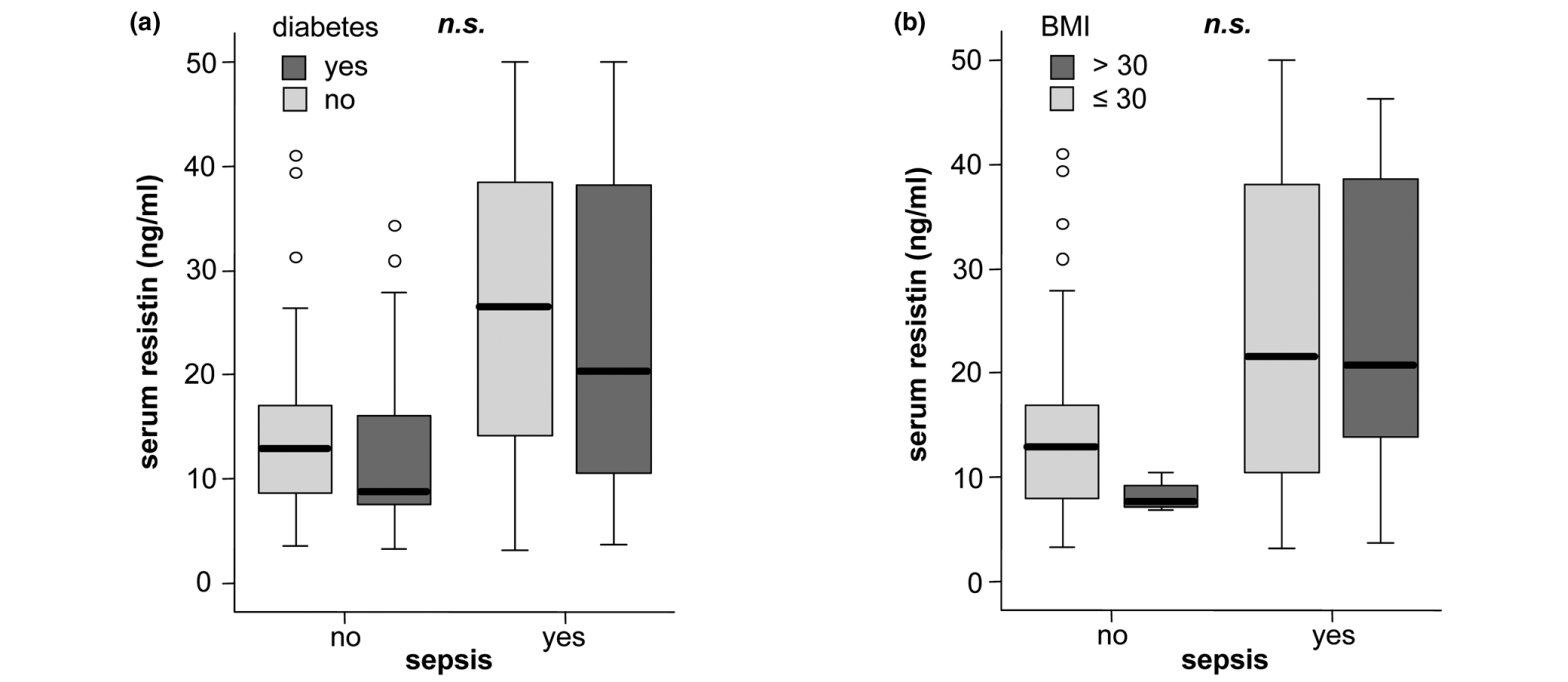
Association of serum resistin with diabetes and obesity in critically ill patients. **(a) **Serum resistin levels do not differ between patients with or without pre-existing diabetes mellitus. **(b) **Serum resistin levels are not associated with obesity as defined by a body mass index of more than 30 kg/m^2^. Box plots are displayed, where the bold black line indicates the median per group, the box represents 50% of the values, and horizontal lines show minimum and maximum values of the calculated non-outlier values; open circles indicate outlier values. ns = not significant.

No significant association between pre-existing diabetes or obesity and serum resistin could be demonstrated (Figure 2).

### Resistin correlates with biomarkers of inflammation, organ function and metabolism

In the cohort of all critical care patients, resistin was found to correlate with a wide number of different biomarkers. The correlating parameters can be classified into markers of inflammation, markers of organ function, and markers of metabolism (Table 4). Serum resistin correlated positively to IL-6 (r = 0.477, *P *< 0.001), IL-10 (r = 0.273, *P *= 0.002), TNF-α (r = 0.509, *P *< 0.001), CRP (r = 0.510, *P *< 0.001), and procalcitonin (r = 0.638, *P *< 0.001). Similar results have been found in the subgroups of septic and non-septic patients, except for the correlation with IL-10, which showed no statistical significance in the group of non-sepsis patients (Table 4).

**Table 4:** Correlations with serum resistin levels

	**All patients**		**Sepsis**		**Non-sepsis**	
			
**Parameters**	**r**	***P***	**r**	***P***	**r**	***P***
***Markers of inflammation***						
IL-6	0.477	< 0.001	0.289	0.004	0.671	< 0.001
IL-10	0.273	0.002	0.231	0.027		ns
TNF-α	0.509	< 0.001	0.419	< 0.001	0.687	< 0.001
CRP	0.510	< 0.001	0.395	< 0.001	0.389	0.012
Procalcitonin	0.638	< 0.001	0.594	< 0.001	0.458	0.003
						
***Markers of organ function***						
Creatinine	0.462	< 0.001	0.420	< 0.001	0.602	< 0.001
Cystatin C	0.442	< 0.001	0.404	< 0.001		ns
Lactate		ns	0.286	0.006		ns
PCHE	-0.269	0.002	-0.280	0.006		ns
Bilirubin	0.221	0.013	0.224	0.035		ns
						
***Markers of metabolism***						
Protein	-0.199	0.02		ns		ns
fT3	-0.319	< 0.001	-0.252	0.016		ns
fT4	-0.276	0.001	-0.229	0.028		ns
Cholesterol	-0.245	0.004	-0.296	0.003		ns
HDL	-0.277	0.002	-0.254	0.019		ns
LDL	-0.359	< 0.001	-0.378	< 0.001		ns
Lp(a)		ns	-0.223	0.040		ns
Glucose	-0.320	< 0.001		ns		ns
Insulin	-0.209	0.02		ns		ns
HOMA IR	0.314	< 0.001		ns		ns
PO_4_	0.321	< 0.001	0.308	0.003	0.417	0.008
Cortisol	0.312	0.001	0.275	0.010		ns
Parathormone	0.212	0.019	0.228	0.033		ns
						
***Clinical scoring***						
APACHE II		ns		ns	0.481	0.005

r = correlation coefficient; r and *P *values by Spearman rank correlation.

APACHE = Acute Physiology and Chronic Health Evaluation;CRP = C-reactive protein; fT3 = free triiodo-thyronine; fT4 = free thyroxine; HDL = high-density lipoprotein; HOMA IR = homeostasis model assessment index of insulin resistance; IL = interleukin; LDL = low-density lipoprotein; Lp(a) = lipoprotein (a); ns = not significant; PCHE = pseudocholinesterase; PO_4 _= phosphate; TNF-α = tumor necrosis factor α.

**Figure 3:**
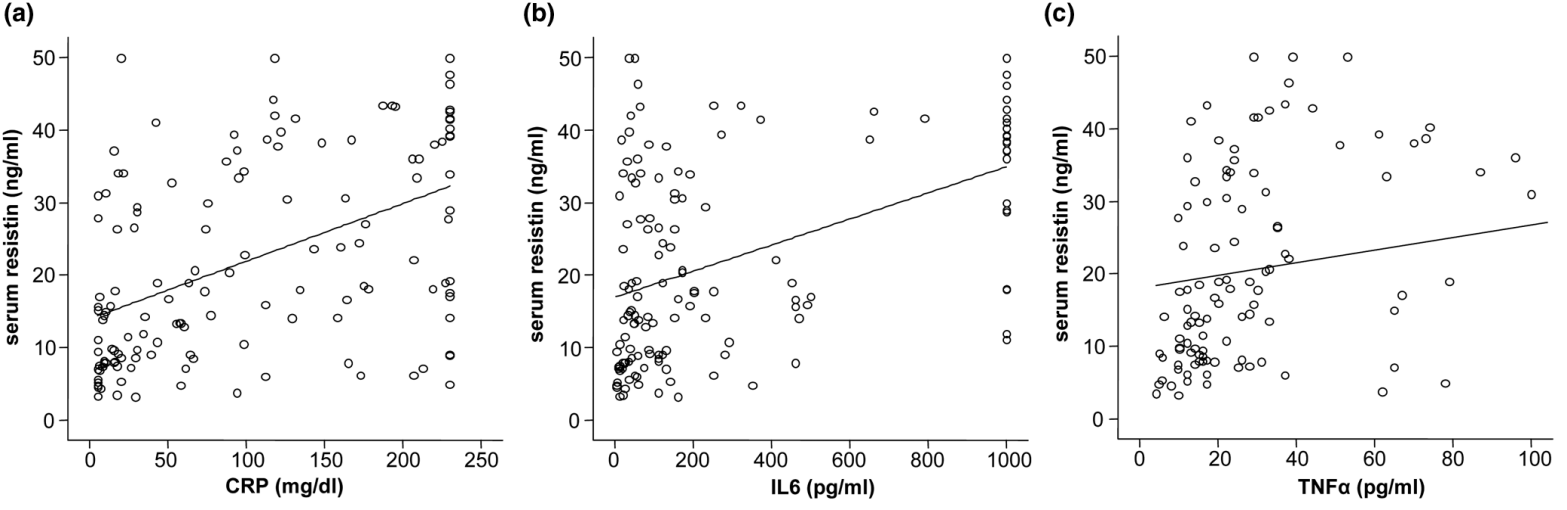
Correlation of serum resistin to biomarkers of inflammation in critically ill patients. Serum resistin is strongly correlated with **(a) **C-reactive protein CRP (r = 0.510, *P *< 0.001), **(b) **IL-6 (r = 0.477, *P *< 0.001), and **(c) **TNF-α (r = 0.509, *P *< 0.001). Spearman rank correlation test.

Renal failure was associated with elevated serum resistin, as resistin correlated with creatinine (r = 0.462, *P *< 0.001) and cystatin C (r = 0.442, *P *< 0.001). Furthermore, hepatic biosynthetic capacity was related to resistin, as parameters indicating diminished hepatic function such as pseudocholinesterase (r = -0.269, *P *= 0.002, Figure 3d) and bilirubin (r = 0.221, *P *= 0.013) correlated with resistin. The correlation with renal function was evident in sepsis and non-sepsis patient subgroups as well, whereas the impact of liver function could only be found in patients with sepsis.

In critically ill patients, hyperinsulinemia and hyperglycemia are common findings and predictive for an unfavorable outcome [3,21]. The mechanisms of insulin resistance in critically ill patients are not well understood; resistin might possibly act as a link between acute inflammation and altered metabolic homeostasis. For the total patient cohort, serum resistin was correlated to insulin resistance as calculated by the HOMA-IR (homeostasis model assessment for insulin resistance) index and inversely correlated with glucose and insulin at admittance prior to intensive care interventions (Table 4). However, these correlations were not observed in the subgroups of sepsis and non-sepsis patients (Table 4). Moreover, markers of lipid metabolism, for example, cholesterol (r = -0.296, *P *= 0.003), high-density lipoprotein (r = -0.254, *P *= 0.019), low-density lipoprotein (r = -0.378, *P *< 0.001) and lipoprotein (A) (r = -0.223, *P *= 0.040) were found to correlate inversely with serum resistin in all critical care patients as well as in the subgroup of sepsis patients.

### Resistin may be a prognostic factor for survival in non-sepsis patients

Cox regression analyses and Kaplan-Meier curves were used to assess the impact of resistin on ICU and overall survival during an almost three-year follow-up among all critical care patients and the subgroups of sepsis and non-sepsis patients. Regarding all ICU patients, no association between survival and resistin serum levels could be revealed (data not shown). No correlation between resistin levels and survival could be demonstrated for sepsis patients either (data not shown).

**Figure 4:**
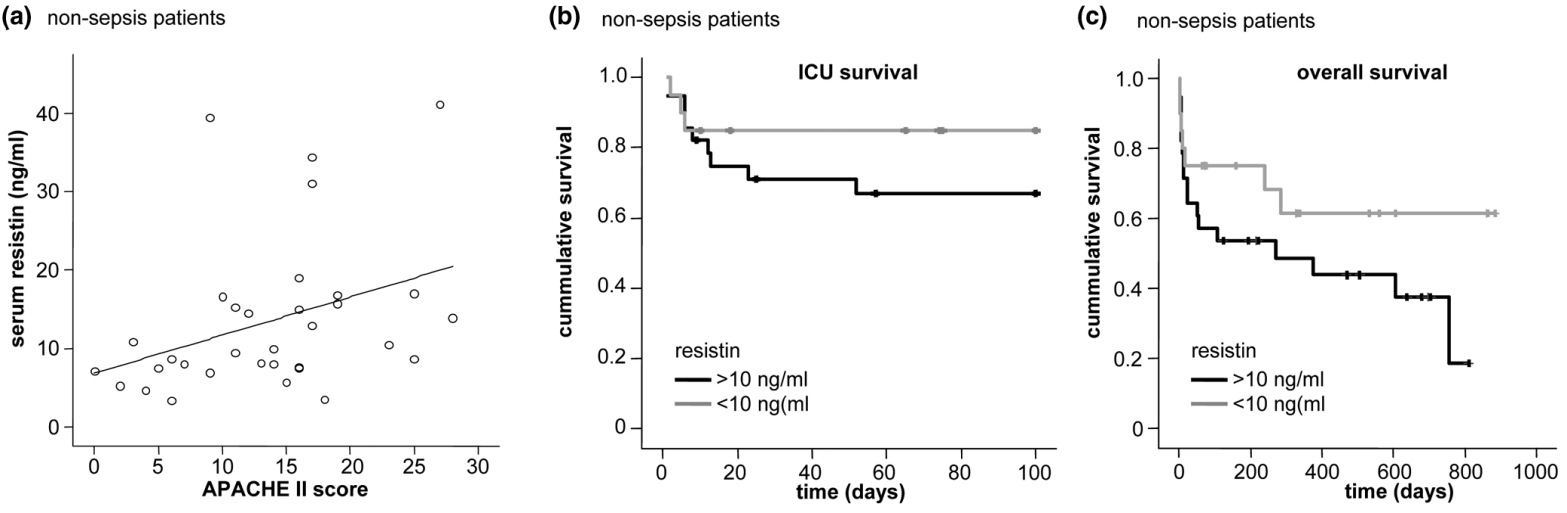
Association of serum resistin with severity of disease and survival in critically ill patients. **(a) **Serum resistin is correlated with Acute Physiology and Chronic Health Evaluation (APACHE) II score (r = 0.481, *P *= 0.005, Spearman rank correlation test) as a marker of severity of disease only in non-sepsis patients (n = 48, shown), but not in sepsis patients (n = 122, not shown). **(b & c) **Serum resistin is a prognostic marker in non-sepsis patients. **(b) **Kaplan-Meier survival curves of non-sepsis patients are displayed, showing that patients with high serum resistin levels (> 10 ng/ml, black) have an increased mortality ain the intensive care unit as compared with patients with low serum resistin (≤ 10 ng/ml, grey). **(c) **Kaplan-Meier survival curves of non-sepsis patients are displayed, showing that patients with high serum resistin levels (> 10 ng/ml, black) have an unfavorable prognosis with respect to overall survival as compared with patients with low serum resistin (≤ 10 ng/ml, grey). Marks on the survival curves represent the times of censored observation.

Remarkably, in patients without sepsis, resistin was correlated with the APACHE II score on admission (r = 0.481, *P *= 0.005, Figure 4a). In these non-sepsis patients, high resistin levels were an adverse prognostic indicator for the ICU (Figure 4b) as well as overall survival (Figure 4c, *P *= 0.046, Cox regression model).

## Discussion

This study emphasizes the role of resistin as an acute-phase protein in critical care circumstances. Compared with healthy volunteers all critical care patients showed elevated resistin levels. Levels were higher in sepsis than in non-sepsis patients with a clear association to markers of the inflammatory response including white blood cell count, CRP, procalcitonin, and with the proinflammatory cytokines IL-6, IL-10, and TNF-α. In recent studies, a correlation between serum resistin and CRP was demonstrated while investigating patients with diabetes [22], coronary artery disease [23,24], or healthy volunteers [25]. Our study now shows that resistin is elevated in states of critical illness, even without evident infection. The clear association between resistin and inflammatory markers also in the non-sepsis patients indicate that resistin is a component of the systemic inflammatory response. In severe sepsis or septic shock resistin concentrations are twice as high as in non-sepsis critically ill patients.

In diabetic or obese subjects, resistin has been shown to be closely correlated to hyperinsulinemia, hyperglycemia, and insulin resistance in several studies [14,26,27]. In contrast, other studies could not verify these findings in insulin-resistant patients or those with type 2 diabetes [28,29]; resistin concentrations in these patients did not correlate to HOMA-IR, BMI, or total cholesterol [15,30]. Regarding inconclusive data from these studies, the endocrinologic role of resistin in human glucose metabolism and insulin resistin, unlike the findings in murine models, is still unclear. In our cohort as well as in a prior study of septic patients [9], resistin did not correlate to obesity measured by BMI in both subgroups of sepsis and non-sepsis patients which suggests that in circumstances of critical illness the release of resistin by macrophages plays a superior role compared with secretion from adipocytes. In line, plasma resistin concentration on admission to the ICU did not correlate to pre-existing diabetes mellitus in the sepsis or non-sepsis patients.

For the subgroups of sepsis and non-sepsis patients, we could not find an association of resistin levels on admittance with hyperinsulinemia and glucose levels. The insulin and glucose values were promptly collected on admission, so they should be unaffected by therapy, for example, insulin, glucose and catecholamine infusions. Likewise, in a recent study resistin levels in critical care patients did not match with glucose, although the authors discussed the affect of therapeutical interventions [9]. However, serum resistin was positively correlated with the HOMA-IR as a marker of insulin resistance. Resistin in critically ill patients therefore seems to contribute to acute inflammatory responses and also to insulin resistance in different ways and to differing degrees.

No association could be shown between resistin levels at admittance and ICU survival or the overall survival of all patients, as well as severity of disease, as measured by APACHE II score for the subgroup of sepsis patients. Remarkably, non-survivors in the subgroup of non-sepsis patients had significantly higher resistin levels than survivors. Assuming that high resistin levels in critical care patients are dependent on macrophageal release in acute inflammation, high resistin levels may indicate an excessive inflammatory reaction, possibly explaining why serum resistin is an independent factor of survival in this cohort. However, we would like to stress that death was not a prospectively defined end-point, and that the results can only be hypothesis generating and require validation in further studies. Our observation that high resistin is a predictor for an unfavorable prognosis only in non-sepsis, but not in sepsis, patients further suggests that the massive acute phase response, as mirrored by elevated resistin, is of considerable harm in the absence of infection. Further studies are warranted to evaluate the potential impact for interventional approaches targeting macrophageal resistin and other cytokine releases in non-septic critically ill patients as well as its clinical value as a novel prognostic biomarker.

Beyond markers of sepsis and inflammation we could demonstrate a strong correlation of serum resistin concentration to various other laboratory parameters. Supporting previous findings, circulating resistin levels are strongly associated with the glomerular filtration rate [31]. For the subgroup of sepsis patients we could demonstrate that resistin is significantly increased in patients with impaired liver function, as evaluated by serum pseudocholinesterase activity and bilirubin concentration, compared with healthy controls. In full agreement, an inverse relation of resistin levels and hepatic biosynthetic capacity in liver cirrhosis has been described [32]. Both observations, correlations with renal and liver dysfunction, are in agreement with the interpretation of serum resistin as a sensitive indicator of the systemic inflammatory response in sepsis.

## Conclusions

Our study demonstrates the potential role of resistin as an acute-phase protein in critically ill patients and its correlation to renal and liver function, and metabolism. Future studies are required to establish if resistin might serve as a novel prognostic biomarker predicting ICU and overall survival in critically ill patients.

## Key messages

• Resistin, a hormone mainly derived from macrophages in humans and from adipose tissue in rodents, has been implicated in glucose metabolism and insulin sensitivity.

• Resistin serum concentrations are elevated in all critical care patients compared with healthy controls and further elevated in patients with sepsis.

• The clear association between serum resistin and inflammatory markers indicate that resistin is a component of the systemic inflammatory response.

• Resistin correlates with renal and liver function as well as with metabolic and endocrine markers.

• Resistin may be a prognostic factor for survival in non-sepsis patients, but not sepsis patients, and could therefore possibly serve as a novel biomarker in critically ill patients.

## Abbreviations

APACHE: Acute Physiology and Chronic Health Evaluation; BMI: body mass index; CRP: C-reactive protein; ELISA: enzyme-linked immunosorbent assay; HOMA-IR: homeostasis model assessment index of insulin resistance; ICU: intensive care unit; IL: interleukin; TNF-α: tumor necrosis factor α.

## Competing interests

The authors declare that they have no competing interests.

## Authors' contributions

AK, FT, and CT designed the study, analyzed data, and wrote the manuscript. OAG performed the resistin and cytokine measurements. ES collected data and assisted in patient recruitment.
